# Valproic Acid Induces Endocytosis-Mediated Doxorubicin Internalization and Shows Synergistic Cytotoxic Effects in Hepatocellular Carcinoma Cells

**DOI:** 10.3390/ijms18051048

**Published:** 2017-05-12

**Authors:** Subbroto Kumar Saha, Yingfu Yin, Kyeongseok Kim, Gwang-Mo Yang, Ahmed Abdal Dayem, Hye Yeon Choi, Ssang-Goo Cho

**Affiliations:** Department of Stem Cell and Regenerative Biotechnology, Incurable Disease Animal Model & Stem Cell Institute (IDASI), Konkuk University, Seoul 05029, Korea; subbroto@konkuk.ac.kr (S.K.S.); yfy_21@hotmail.com (Y.Y.); proproggs@naver.com (K.K.) slayersgod@nate.com (G.-M.Y.); ahmed_morsy86@yahoo.com (A.A.D.); hyeon.choi24@gmail.com (H.Y.C.)

**Keywords:** valproic acid, doxorubicin, reactive oxygen species, autophagy, cell death, caveolae endocytosis pathway

## Abstract

Valproic acid (VPA), a well-known histone deacetylase (HDAC) inhibitor, is used as an anti-cancer drug for various cancers, but the synergistic anti-cancer effect of VPA and doxorubicin (DOX) combination treatment and its potential underlying mechanism in hepatocellular carcinoma (HCC) remain to be elucidated. Here, we evaluate the mono- and combination-therapy effects of VPA and DOX in HCC and identify a specific and efficient, synergistic anti-proliferative effect of the VPA and DOX combination in HCC cells, especially HepG2 cells; this effect was not apparent in MIHA cells, a normal hepatocyte cell line. The calculation of the coefficient of drug interaction confirmed the significant synergistic effect of the combination treatment. Concurrently, the synergistic apoptotic cell death caused by the VPA and DOX combination treatment was confirmed by Hoechst nuclear staining and Western blot analysis of caspase-3 and poly (ADP-ribose) polymerase (PARP) activation. Co-treatment with VPA and DOX enhanced reactive oxygen species (ROS) generation and autophagy, which were clearly attenuated by ROS and autophagy inhibitors, respectively. Furthermore, as an indication of the mechanism underlying the synergistic effect, we observed that DOX internalization, which was induced in the VPA and DOX combination-treated group, occurred via by the caveolae-mediated endocytosis pathway. Taken together, our study uncovered the potential effect of the VPA and DOX combination treatment with regard to cell death, including induction of cellular ROS, autophagy, and the caveolae-mediated endocytosis pathway. Therefore, these results present novel implications in drug delivery research for the treatment of HCC.

## 1. Introduction

According to the World Health Organization (WHO), hepatocellular carcinoma (HCC) is the second most common cause of cancer-related deaths worldwide. Conventional therapeutics for HCC, including chemotherapy and radiotherapy, have very limited efficacy [1]. HCC has hypervascular characteristics and, thus, a standard treatment such as transarterial chemoembolization (TACE) is feasible [2]. However, locally recurrent nodules or advanced infiltrative HCCs may occur infrequently after TACE treatment; in these cases, TACE or systemic chemotherapy is ineffective for the treatment of HCC. Thus, there is an urgent need to develop an alternative HCC therapy.

Doxorubicin (DOX) is a well-known, potent drug for the treatment of breast cancer patients [3], even though it displays adverse side-effects in terms of cardiotoxicity, hepatotoxicity nephrotoxicity, typhlitis, and other toxicities [4,5]. For advanced HCC treatment, doxorubicin (DOX), an anthracycline anticancer agent, has also been used frequently; however, it shows a maximum response rate of approximately 15–20% [6,7], which is not sufficient for HCC treatment. Previous studies have suggested a number of factors that might cause DOX resistance in HCC [8,9,10]. Moreover, an in vitro study showed that doxorubicin resistance was more severe under hypoxic conditions compared to normoxic conditions in HCC cells [11]. DOX was reported to induce the cellular oxidative stress, which prevents cancer cells and augments an inflammatory microenvironment, with a common unwanted cytotoxicity [12]; thus, DOX mono-treatment is not a desirable drug. For more efficient cancer treatment, a combination treatment of DOX with other anticancer drugs may be an effective approach to HCC therapy.

Valproic acid (VPA), a potent and specific histone deacetylase (HDAC) inhibitor, is another established and widely used antiepileptic drug that has been employed for the treatment of epileptic seizures [13]. Moreover, it has been used as an anticonvulsant and a mood-stabilizing drug, similar to lithium, and also has neuroprotective effects in neurodegenerative conditions [14,15,16,17]. VPA was recognized as a hepatotoxic drug [18,19]; additionally, several studies have demonstrated that VPA treatment led to growth inhibition or apoptosis or both in a range of cancer cells [20,21,22,23], including HCC cells [24,25,26]. As a HDAC inhibitor, VPA can prompt the differentiation of various cancer cells in vitro and prevent tumor progression and metastasis in vivo [27]. VPA was also reported to modulate the cellular membrane trafficking, the process by which proteins and other macromolecules are distributed throughout the cell [28,29,30]. Although membrane trafficking requires the coordination of multiple signaling events to control cargo sorting and processing, and endosome maturation and VPA were revealed to have a role at the some step of membrane trafficking, including the fusion of Golgi-derived vesicles with the pre-vacuolar compartment [28,31,32], the exact mechanism for VPA-mediated modulation of the endocytosis pathway remains to be elucidated.

Membrane trafficking uses membrane-bound vesicles as transport intermediaries and cargo molecules are enclosed within or associate with the membrane of the vesicles [33]. Endocytosis, the internalization of extracellular materials, is one of the promising membrane trafficking processes that is critical for the normal function and survival of eukaryotic cells [34]. Endocytic entry of protein and lipid molecules may be the first step in endosomal trafficking, followed by the formation of early endosomes and then late endosomes [34]. Endocytic trafficking involves a series of steps including endocytosis, cargo sorting and processing, intracellular membrane fusion and fission, vesicle mobility, and exocytosis [35]; the endocytic pathways include clathrin-dependent endocytosis, caveolae-dependent endocytosis, macropinocytosis, and phagocytosis. Recently, the pathways of clathrin and caveolae receptor-mediated endocytosis were well studied in cellular membrane trafficking for cancer therapy [36,37,38,39].

Previously, several studies depicted that nanoparticle-bound DOX could internalize into cells by receptor-mediated endocytic pathways and stimulate cytotoxic pathways in various cancers [40,41,42]. VPA was reported to be able to conjugate with various potential drugs, such as epilepsy drugs [43], and may play a crucial role in membrane trafficking regarding drug delivery.

In this study, we assessed the combination treatment of VPA and DOX in HCC and attempted to determine the underlying mechanisms of the synergistic effect of VPA and DOX and the VPA-mediated internalization of DOX in HepG2 cells.

## 2. Results

### 2.1. Combination Treatment of VPA and DOX Synergistically Inhibits the Viability of Human HCC Cells

**Figure 1:**
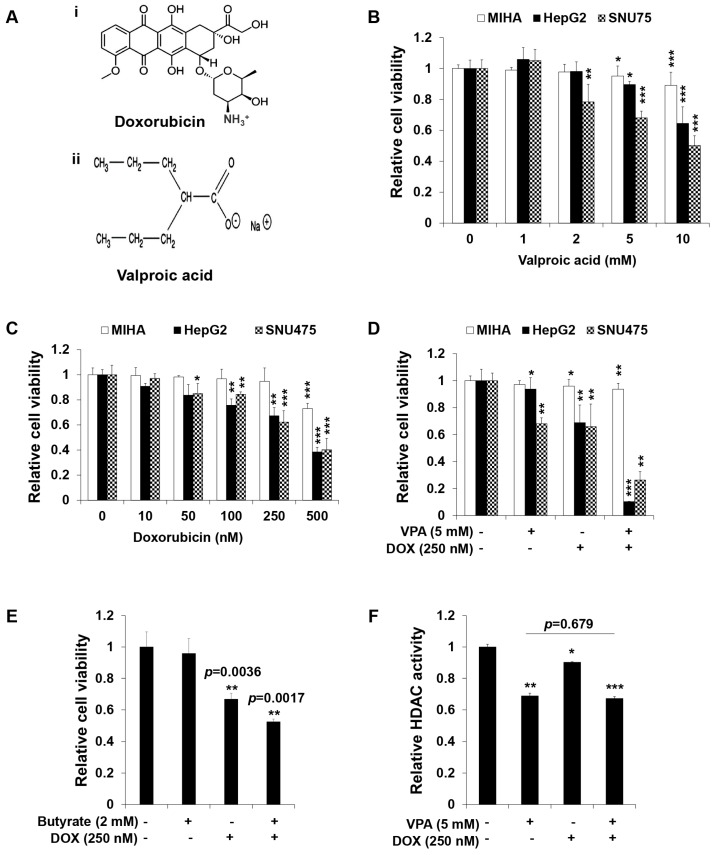
The combination treatment of valproic acid (VPA) and doxorubicin (DOX) synergistically inhibited the viability of hepatocellular carcinoma (HCC) cells. (**A**) Structure of DOX (**i**) and VPA (**ii**); (**B**) the viability of MIHA, HepG2, and SNU475 cells was determined by EZ-Cytox assay after 48-h exposure to the indicated concentration of VPA; (**C**) the viability of MIHA, HepG2, and SNU475 cells was determined by EZ-Cytox assay after 48-h exposure to the indicated concentration of DOX; (**D**) the viability of MIHA, HepG2, and SNU475 cells was determined by EZ-Cytox assay after 48-h exposure to the indicated concentration of VPA and DOX monotherapies and combination treatment; (**E**) monotherapy and combination treatment of DOX and butyrate at the indicated concentration was used to determine HepG2 cell viability after 48-h exposure using EZ-Cytox assay; (**F**) the HDAC activity of HepG2 cells was assessed using a colorimetric HDAC activity assay after 48-h exposure to the indicated concentration of VPA and DOX. Three independent experiments were performed and results reported as the mean ± standard deviation (SD). * *p* < 0.05, ** *p* < 0.01, *** *p* < 0.001 compared with the control group.

Normal hepatocytes (MIHA) and HCC cells (HepG2 and SNU475) were separately treated with VPA or DOX for 48 h. The results showed that HCC cell viability was inhibited significantly in a dose-dependent manner, while little or no effect was observed in MIHA cells (Figure 1B,C). Furthermore, to determine the synergistic dose of VPA and DOX, we calculated the coefficient of drug interaction (CDI) for a single dose of VPA (5 mM) and different doses of DOX in HepG2 cells (Table 1) [44], which revealed that the combination of 5 mM VPA and 250 nM DOX generated the lowest CDI value and therefore showed the greatest synergistic effect on the viability of HepG2 cells. Hence, MIHA, HepG2, and SNU475 cells were treated with VPA (5 mM) and DOX (250 nM) for 48 h. The combination treatment showed a significantly synergistic cytotoxic effect (approximately 90%) in HCC cells, especially HepG2 cells (*p* < 0.001), whereas no synergy, or a lower synergistic effect, was observed in MIHA cells (Figure 1D). As VPA is an HDAC inhibitor (HDI), we assessed the effect of a different HDI, 2 mM sodium butyrate [45], on the viability of HepG2 cells. Sodium butyrate did not demonstrate any synergistic effect with DOX in HepG2 cells (Figure 1E). We also performed HDAC activity assay and revealed that HDAC activity was expectedly attenuated by the VPA treatment, while the combination of VPA and DOX treatment did not show a significant (*p* = 0.679) reduction compared to only VPA treatment did (Figure 1F). In addition, only DOX treatment showed a slight decline in HDAC activity (Figure 1F). Therefore, VPA is suggested to exhibit an HDAC-independent synergistic effect with DOX on the viability of HepG2 HCC cells.

### 2.2. Combination Treatment of VPA and DOX Synergistically Induces Apoptotic Cell Death in HepG2 Cells

**Figure 2:**
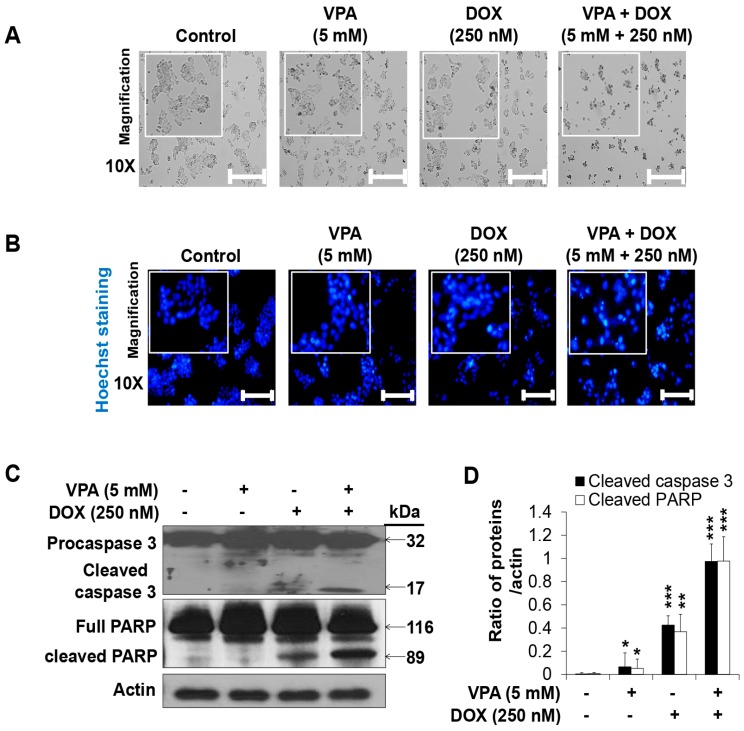
The combination treatment of valproic acid (VPA) and doxorubicin (DOX) synergistically induced apoptosis of HepG2 cells. (**A**) Morphology of HepG2 cells treated with monotherapies and combination treatment of VPA and DOX at indicated concentration after 48-h treatment. Images were taken using phase contrast inverted light microscopy. Scale bar represents 200 μm; (**B**) Hoechst nuclear staining was used to detect apoptosis with condensed and fragmented nucleus in HepG2 cells after 48-h incubation with the indicated concentration of VPA and DOX monotherapies and the combination treatment. Images were taken using fluorescence inverted microscopy. Scale bar represents 200 μm; (**C**) Levels of pro- and cleaved-caspase3 and full length-and cleaved-PARP were analyzed in the indicated treated cells by using Western blotting. Actin was used as the loading control; (**D**) The intensity of cleaved-caspase3 and cleaved-PARP bands were quantified by scanning densitometry program ImageJ and normalized to that of actin. At least three independent experiments were performed and results shown as the mean ± standard deviation (SD). * *p* < 0.05, ** *p* < 0.01, *** *p* < 0.001 compared with the control group.

The VPA and DOX combination treatment led to more severe changes in cell morphology (Figure 2A) than that observed for treatment with the individual drugs. Next, we conducted Hoechst nuclear staining and revealed that apoptotic nuclear condensation and fragmentation significantly increased upon the VPA and DOX combination treatment in HepG2 cells in comparison with that reported for the monotherapies (Figure 2B). In addition, cleaved caspase-3 and PARP cleavage increased significantly in the combination-treated group while VPA or DOX alone had no effect or only a slight effect (Figure 2C,D), which confirmed the synergistic cytotoxicity of the VPA and DOX combination treatment in HCC.

### 2.3. Combination Treatment of VPA and DOX Synergistically Induces ROS and Autophagy Generation in HepG2 Cells

**Figure 3:**
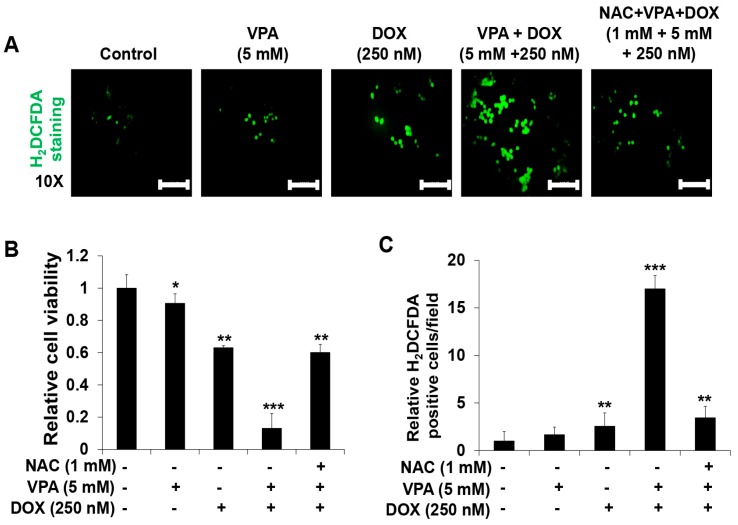
Combination treatment of valproic acid (VPA) and doxorubicin (DOX) synergistically enhanced reactive oxygen species (ROS) generation in HepG2 cells. (**A**) The 2′,7′-dichlorofluorescein diacetate (H_2_DCFDA) fluorescence probe was used to determine ROS generation in HepG2 cells at the indicated concentrations of VPA and DOX monotherapies and combination treatment after incubation for 48 h. Images were taken using fluorescence inverted microscopy. Scale bar represents 200 μm; (**B**) The viability of HepG2 cells was determined after 48-h incubation at the indicated experimental condition by using EZ-Cytox assay; (**C**) the ROS-generating cells were counted in different fields (containing at least 40 cells per field) and calculated relative to the control group for each experimental condition. Three independent experiments were performed and results shown as the mean ± standard deviation (SD). * *p* < 0.05, ** *p* < 0.01, *** *p* < 0.001 compared with the control group.

The VPA and DOX combination treatment led to an increased ROS generation (Figure 3A) compared with that reported for treatment with the individual drugs. We also found that the addition of *N*-acetylcysteine (NAC) (1 mM), a ROS scavenger, suppressed the synergistic induction of apoptosis (Figure 3B) and ROS generation (Figure 3C) in HepG2 cells, which indicated that the VPA and DOX combination treatment might induce synergistic cytotoxicity through the modulation of ROS generation.

**Figure 4:**
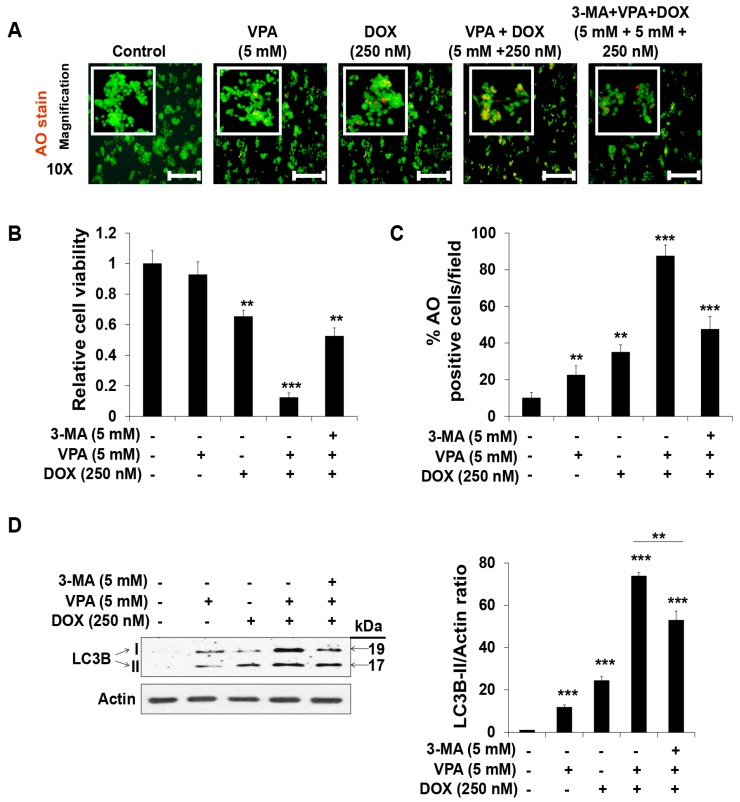
Combination treatment of valproic acid (VPA) and doxorubicin (DOX) synergistically augmented the autophagy of HepG2 cells. (**A**) Acridine orange (AO) staining was used to detect acidic vesicles in HepG2 cells at the indicated concentration of VPA and DOX monotherapies and combination treatment after incubation for 48 h. Images were taken using fluorescence inverted microscopy. Red color represents acidic vesicle and green color represents non-acidic vesicle. Scale bar represents 200 μm; (**B**) The viability of HepG2 cells was analyzed after 48-h incubation in the indicated experimental condition by using EZ-Cytox assay; (**C**) Percentages (%) of AO-positive cells were counted in different fields (containing at least 40 cells per field); (**D**) LC3 I and II protein levels were analyzed using Western blotting. Actin was used as the loading control. The intensity of LC3B-II bands was quantified by scanning densitometry program ImageJ and normalized to that of actin (right panel). Three independent experiments were performed and results reported as the mean ± standard deviation (SD). ** *p* < 0.01, *** *p* < 0.001 compared with the control group.

To determine the effect of the VPA and DOX combination treatment on autophagy, we used the acridine orange (AO) staining method and found that the number of acidic organelles significantly increased following the VPA and DOX combination treatment, while treatment with either VPA or DOX alone led to very slight AO staining (Figure 4A–C). Additionally, we found that pre-incubation with 3-methyladenine (3-MA), an autophagy inhibitor, led to an apparent decrease in the synergistic induction of apoptosis (Figure 4B) and autophagy generation (Figure 4C) by the VPA and DOX combination treatment in HepG2 cells. Moreover, the amount of LC3B-II protein, an autophagy biomarker, was significantly augmented upon VPA- or DOX-alone treatment and more dramatically upon VPA and DOX combination treatment, whereas pre-treatment of 3-MA significantly relieved the VPA and DOX combination treatment effect (Figure 4D), which suggested that the combination treatment might exert a potential synergistic cytotoxic effect by regulating the autophagy pathway.

### 2.4. VPA Induces Internalization of DOX in HepG2 Cells through Caveolae-Mediated Endocytosis

**Figure 5:**
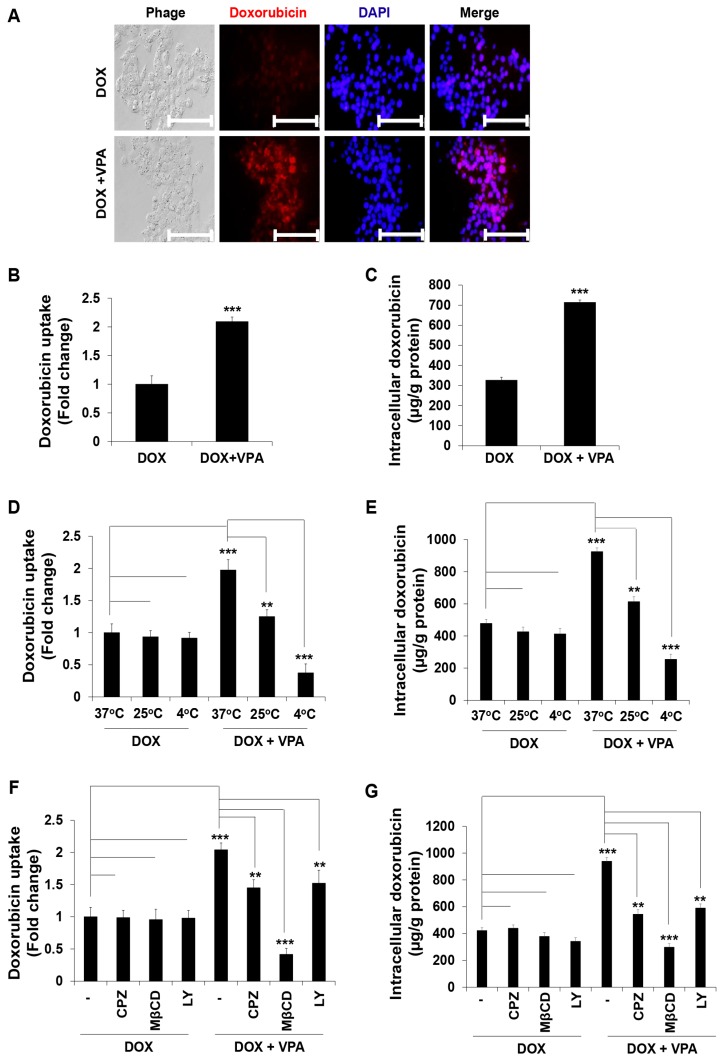
Valproic acid (VPA) induces cellular doxorubicin (DOX) internalization and mediates the caveolae endocytosis pathway in HepG2 cells. (**A**) Cellular DOX internalization images were captured of the indicated treated cells using fluorescence inverted microscopy. Scale bar represents 200 μm; (**B**) DOX uptake was measured in the indicated treated cells at excitation and emission wavelengths of 470 and 570 nm, respectively, using a spectrofluorometer; (**C**) intracellular DOX concentration was measured in the indicated treated cells at excitation and emission wavelengths of 470 and 570 nm, respectively, using a spectrofluorometer; (**D**,**E**) DOX uptake and intracellular DOX concentration were measured in the indicated treated cells at the indicated temperature by using a spectrofluorometer; (**F**,**G**) DOX uptake and intracellular DOX concentration were measured in indicated treated cells cultured at 37 °C with pre-incubated different endocytosis pathway inhibitors (CPZ, 10 μM; MβCD, 3 mM; and LY, 20 μM) using a spectrofluorometer. Fluorescence intensity was measured at the excitation and emission wavelengths of 470 and 570 nm, respectively. Three independent experiments were performed and shown as the mean ± standard deviation (SD). ** *p* < 0.01, *** *p* < 0.001 compared with the control group.

To understand the mechanism underlying the synergistic effect of the VPA and DOX combination treatment, we measured the cellular internalization of DOX by evaluating the fluorescence of the internalized DOX. We found that DOX internalization increased significantly upon the VPA and DOX combination treatment compared to that observed with DOX alone (Figure 5A,B). Then, we quantified the intracellular DOX concentration using a fluorescence plate-reader and confirmed that the intracellular DOX concentration markedly increased following the VPA and DOX combination treatment (Figure 5C).

It was previously reported that, although free DOX can internalize into cells through diffusion [46], it may also enter cells through cellular surface receptors by conjugation with other chemicals or particles [46,47]. As the prominent uptake pathway of extracellular materials into the cell is endocytosis [48,49], we hypothesized that VPA might interact with DOX, forming a VPA-DOX complex, which may internalize into the cells through the endocytosis pathway. Therefore, we cultured cells at different temperatures (37, 25, and 4 °C) and investigated the level of DOX internalization. It has been established that the endocytosis pathway may be retarded or stopped at 4 °C [50]. After the cells were cultured for 3 h in the presence of DOX alone or the VPA and DOX combination, they were analyzed using a spectrofluorometer. The results showed that DOX internalization upon the VPA and DOX combination treatment drastically decreased following culture at 25 and 4 °C compared to that observed at 37 °C (Figure 5D). Quantification of the internalized DOX also revealed that VPA-mediated internalization of DOX significantly decreased at a low temperature (Figure 5E).

Subsequently, we tried to clarify which endocytosis pathway was responsible for this phenomenon and used three different receptor-mediated endocytosis pathway inhibitors: chlorpromazine (CPZ), methyl-β-cyclodextrin (MβCD), and LY294002 (LY). We observed that DOX internalization was suppressed by MβCD pre-treatment compared to that observed with the other inhibitors (Figure 5F). The intracellular DOX concentration was confirmed to be dramatically decreased upon MβCD pre-treatment (Figure 5G), which strongly indicated that VPA might enhance DOX internalization into the cell predominantly through the caveolae-mediated endocytosis pathway.

**Figure 6:**
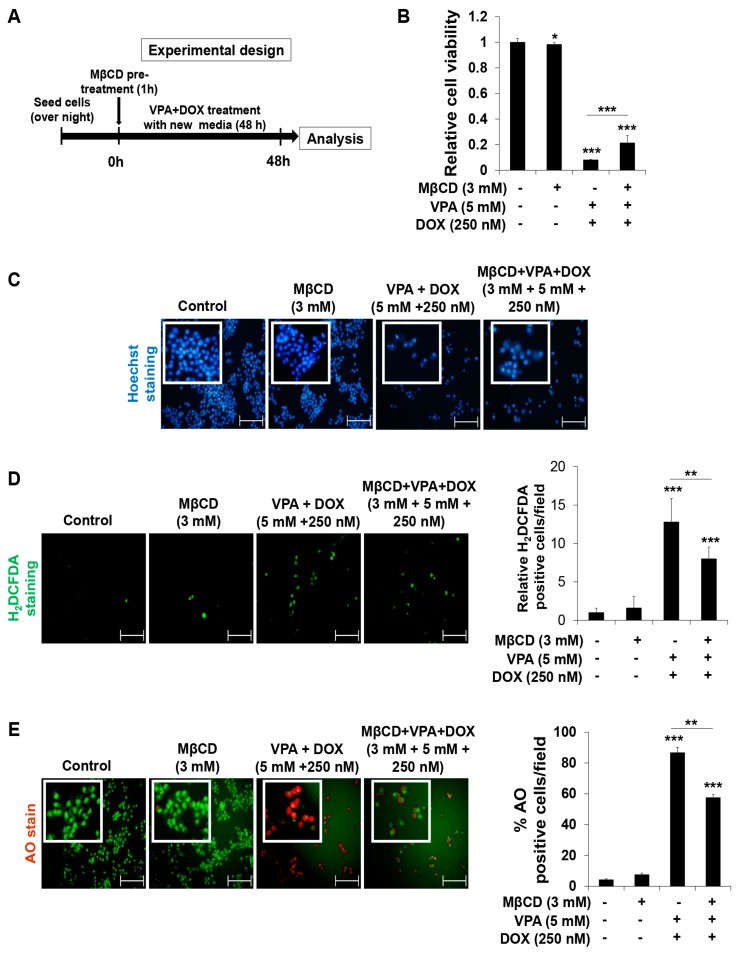
Pre-treatment of MβCD significantly recovers the effect of VPA and DOX combination treatment in HepG2 cells. (**A**) The experimental design of MβCD pre-treatment with or without VPA-DOX combined treatment in HepG2 cells; (**B**) the viability of HepG2 cells was determined at the indicated experimental condition by using EZ-Cytox assay; (**C**) Hoechst nuclear staining was used to detect apoptosis with condensed and fragmented nucleus in HepG2 cells at the indicated experimental condition. Images were taken using fluorescence inverted microscope. Scale bar represents 200 μm; (**D**) The 2′,7′-dichlorofluorescein diacetate (H_2_DCFDA) fluorescence probe was used to determine ROS generation in HepG2 cells at the indicated experimental condition. Images were taken using fluorescence inverted microscope. Scale bar represents 200 μm. The ROS-generating cells were counted in different fields (containing at least 40 cells per field) and calculated relative to the control group for each experimental condition (right panel); (**E**) Acridine orange (AO) staining was used to detect acidic vesicles in HepG2 cells at the indicated experimental condition. Images were taken using fluorescence inverted microscope. Red color represents acidic vesicle and green color represents non-acidic vesicle. Scale bar represents 200 μm. Percentages (%) of AO-positive cells were counted in different fields (containing at least 40 cells per field) (right panel). Three independent experiments were performed and results reported as the mean ± standard deviation (SD). * *p* < 0.05, ** *p* < 0.01, *** *p* < 0.001 compared with the control group.

Next, we tried to evaluate the effect of MβCD pre-treatment with or without VPA and DOX combined treatment in HepG2 cells (Figure 6A). We found that MβCD pre-treatment showed a negligible effect on cell viability (Figure 6B) and the VPA and DOX combined treatment-induced decrease of cell viability was significantly relieved by MβCD pre-treatment (Figure 6B). MβCD pre-treatment also did not induce any apparent effect on apoptosis (Figure 6C), intracellular ROS generation (Figure 6D), or autophagy (Figure 6E). Significantly, MβCD pre-treatment resulted in apparent recovery from the VPA and DOX combination treatment-mediated effect on apoptosis (Figure 6C), intracellular ROS generation (Figure 6D), and autophagy (Figure 6E), confirming that the caveolae-mediated endocytosis pathway makes important role in the VPA-mediated DOX internalization into the cell and the synergistic anti-cancer effect of VPA and DOX combination treatment.

## 3. Discussion

HCC treatments using traditional radio- and chemotherapies are sometimes inefficient, partly because of their severe hepatotoxicity. In our study, we described the specific and efficient, synergistic anti-proliferative and apoptotic effect of the VPA and DOX combination in HCC cells, especially HepG2 cells (Figure 1 and Figure 2). Recent studies have stated that the combination treatment of FDA-approved anti-HCC drugs such as DOX, sorafenib, cisplatin, interferon α-2b, and fluorouracil could be safely used for HCC patients [6,7,51], but the combination treatment showed a limited response rate (approximately 15–20%). Although DOX, as well as sorafenib, were shown to cause cell death, partially by enhancing apoptosis in HCC cells [6,7,51], the exact mechanism underlying the pharmacological synergy has not yet been determined. Moreover, VPA was reported to sensitize anaplastic thyroid carcinoma (ATC) cells to DOX, which caused apoptosis via the induction of histone hyperacetylation or apoptosis-related gene expression [52,53,54]. Concurrently, several studies demonstrated that VPA showed synergistic effects with well-known anticancer drugs, such as aspirin, flavopiridol, mitomycin C, cisplatin, adriamycin, and DOX, and could induce cell death in various cancer cells [24,52,55,56]. The synergistic anticancer effect of VPA with other drugs was primarily considered to occur through histone acetylation and alteration of apoptosis related gene expression, but the underlying mechanisms of the synergistic effect and drug internalization into the cell remain unknown. In our study, calculations of the CDI confirmed the dramatically significant synergistic effect of the VPA and DOX combination, specifically in HepG2 cells (Table 1). Moreover, we revealed that VPA might exert an HDAC-independent synergistic effect with DOX on the viability of HepG2 cells. As the mechanism underlying the synergistic effect, we observed that DOX internalization, which was induced by the VPA and DOX combination treatment, occurred via the caveolae-mediated endocytosis pathway. We believe this presents novel implications for drug delivery research into the treatment of HCC.

Hoechst nuclear staining and Western blot analysis of caspase-3 and PARP activation confirmed the synergistic apoptotic cell death induced by the VPA and DOX combination treatment (Figure 2). Moreover, the combination treatment resulted in an increased ROS generation and autophagy, which were clearly attenuated by ROS and autophagy inhibitors, respectively (Figure 3 and Figure 4). Oxidative stress and autophagy have been shown to cause cell death in various types of cancers [57,58,59,60]. A previous study demonstrated that the apoptosis of solid tumor and leukemia cells was induced by the generation of ROS following treatment with an HDAC inhibitor [57]. VPA, a well-known HDAC inhibitor, induced ROS generation in several cancer cells, which was attenuated by NAC treatment [58,61]. Concurrently, it enhanced oxidative stress in cells by increasing glutathione (GSH) levels [58]; this supported the involvement of HDAC inhibitor-mediated oxidative stress in anticancer treatment. However, our study revealed that VPA might have an HDAC-independent synergistic effect with DOX on the viability of HepG2 cells (Figure 1).

Several studies demonstrated that autophagy was induced by VPA treatment through the downregulation of the AKT/mTOR pathway in prostate cancer [62]. Moreover, it increased autophagy-mediated lymphoma cell chemo-sensitivity through IP3-mediated PRKAA activation, which was HDAC-independent [59]. Thus, we aimed to investigate whether VPA and DOX monotherapies and the combination treatment induced autophagy in HepG2 cells. AO staining is an established method for the detection of acidic compartments/vesicles in the cell cytoplasm [63]. Our observation in the cells treated with the monotherapy was consistent with previously described studies [59,60]. In addition, the synergistic effect observed with the combination treatment led to marked changes in cell morphology and the formation of acidic vesicles; this effect was diminished by pre-treatment of the cells with 3-MA (Figure 4), which suggested that the synergistic effect on cell death by VPA and DOX monotherapies and the combination treatment might result, at least partially, from the induction of autophagy.

Recently, mono- or combination-treatments of VPA have been used in several types of cancer and showed anti-proliferative activity in both modes [24,55,56]. Importantly, for epilepsy patients, VPA exhibited a significant anti-proliferative activity at clinically pertinent concentrations in the presence of serum at a daily dose of 20–30 mg/kg [64,65]. Our results revealed that the combination treatment of VPA and DOX exhibited a dramatic synergistic effect over VPA or DOX monotherapy at a clinically relevant dose. A number of consistent findings supported that co-treatment of VPA with DOX and paclitaxel (PAX) enhanced the effect of DOX and PAX [66,67,68,69]. Co-treatment of VPA with DOX led to synergistic suppression of cell viability with an increase in caspase-3 activity and CDKN1A, CCNE1, PARP1, and PARP3 proteins expression in ovarian cancer cell lines [67]. In addition, the VPA prodrugs promoted the anti-cancer efficacy of DOX, while a reduced Dox cytotoxicity was observed in non-cancerous cells [68]. Furthermore, Ververis et al. [69] showed another similar finding in cardiomyocytes that combination treatment of different HDAC inhibitors including VPA augmented the DOX-induced DNA double strand breaks, suggesting the positive interaction between VPA and DOX. These findings suggest the positive interaction between VPA and DOX and consequently led to increased cytotoxicity. However, besides HDAC inhibitory activity, VPA can also modulate the cell membrane trafficking such as endocytosis, exocytosis or lipid droplet formation into the cells [28,29,30]. According to Miyatake et al. [28], VPA showed a potential function in membrane trafficking in fission yeast. As a molecular basis for the VPA-mediated modulation of membrane trafficking, vacuolar protein sorting 45 homolog (*vps45*) was identified by a genetic screen method for fission yeast mutants, whereby a gene mutant, vacuolar protein sorting 45+ homolog (*vps45+*) encoded a member of the Sec1/Munc18 family [28]. The *vps45+* mutant along with other mutants including *ypt3+* and *ryh1+* augmented VPA hypersensitivity which led to induction of vacuolar fragmentation and impairment of the glycosylation and secretion of acid phosphatase, consequently prompting membrane trafficking [28]. Moreover, VPA treatment enhanced the cell sensitivity to the cell-wall-digesting enzymes which led to modulation of the membrane trafficking [28]. However, a controversial finding was also reported to state that VPA could impair the signal-induced translocation of PH_Crac_-green fluorescent protein from cytosol to membrane, suggesting the inhibition of phosphatidylinositol-(3,4,5)-trisphosphate (PIP3) production [29]. For the inhibition of PIP3 production, VPA acutely reduced the PIP3-dependent endocytosis and exocytosis [29]. Importantly, a recent study depicted that VPA could augment the accumulation of lipid droplet along with fatty acids and non-polar lipids in hepatocyte and that was independent on VPA-catalyzed teratogenicity and inositol depletion [30], which may suggest a VPA-mediated modulation of lipid rafts endocytosis pathway. As VPA is an epilepsy drug, it may conjugate with various potent drugs, similar to other epilepsy drugs [43]. Freely available DOX can be imported to the cell by diffusion methods [46], while DOX conjugated with other chemicals or particles could pass into the cells via a cellular surface receptor [46,47]. To explain the above studies, we hypothesized that VPA interacted with DOX and formed a particle or complex chemical-like structure, which might be the cause of increased DOX internalization. Thus, we evaluated different cellular surface receptor-mediated pathways: the clathrin-, caveolae-, and macropinocytosis-mediated DOX internalization pathways [47,70,71]. As expected, we observed that DOX internalization increased drastically in the VPA-DOX combination group compared with that in the free DOX group (see Figure 5A–C). Specifically, in the current study, we found that pre-treatment with different inhibitors of cellular surface receptor-mediated endocytosis had a different effect on DOX internalization (see Figure 5D–F). Specifically, MβCD (a caveolae-mediated endocytosis inhibitor) exhibited a dramatic inhibition of DOX internalization among the other inhibitors (Figure 5).

Cyclodextrins (CDs) are non-reducing cyclic glucose oligosaccharides which are good chelators and have a very high affinity for sterols [72]. MβCD is more efficient chelator than other CDs which is employed for the preparation of cholesterol-free solution. MβCD is also employed to disrupt lipid rafts by removing cholesterol from membranes and interrupt endocytosis [73]. We found that MβCD pre-treatment did not show any adverse effect on cell viability, ROS generation, autophagy, and apoptosis, which is consistent with the previous study [74]. Importantly, MβCD pre-treatment significantly recovered the VPA and DOX combination effect, confirming that the synergistic effect of the VPA and DOX combination treatment might be regulated mainly through the caveolae-mediated endocytosis pathway, which consequently induced ROS and autophagy-mediated cell death. Further studies are needed to reveal the synergistic effect of the VPA and DOX combination treatment with regard to the endocytosis-mediated DOX internalization pathway.

## 4. Materials and Methods

### 4.1. Cell Culture and Reagents

The human normal hepatocyte cell line MIHA and the HCC cell line SNU475 were kindly gifted to us by Professor Suk Woo Nam (The Catholic University, Seoul, Korea) and the HCC cell line HepG2 was purchased from ATCC (Manassas, VA, USA). All cell lines used in this study were grown in Roswell Park Memorial Institute (RPMI)-1640 or Dulbecco’s modified Eagle’s medium (DMEM)-high glucose media (Sigma-Aldrich, St. Louis, MO, USA) supplemented with 10% fetal bovine serum (FBS) (Hyclone, Logan, UT, USA) and 1% penicillin-streptomycin (Invitrogen, Carlsbad, CA, USA). The cells were incubated in humidified conditions with 5% CO_2_ at 37 °C. Mycoplasma contamination of all cell lines was tested using BioMycoX^®^ Mycoplasma PCR Detection Kit (Cellsafe, Suwon, Korea) and short tandem repeat (STR) profiling was performed for authentication.

Valproic acid sodium salt (VPA) (Figure 1A(i)), doxorubicin hydrochloride (DOX) (Figure 1A(ii)), sodium butyrate, *N*-acetylcysteine (NAC), 3-methyladenine (3-MA), chlorpromazine (CPZ), methyl-β-cyclodextrin (MβCD), LY 294002 (LY), Hoechst 33258, and acridine orange hydrochloride hydrate (AO) were acquired from Sigma-Aldrich. 2′,7′-Dichlorodihydrofluorescein diacetate (H_2_DCFDA) was purchased from Molecular Probes™ (Eugene, OR, USA).

### 4.2. Cell Viability Assay

MIHA, HepG2, and SNU475 cells (1 × 10^4^ cells/well) were seeded in 96-well plates and grown overnight to confluence. The cells were then treated with the indicated dose of VPA, DOX, and the VPA and DOX combination for 48 h at 37 °C in an atmosphere of 5% CO_2_. After incubation for 48 h, the medium was exchanged with a fresh medium containing EZ-Cytox (Daeil Lab Service, Seoul, Korea) and incubated for an additional 3–4 h at 37 °C in an atmosphere of 5% CO_2_. The absorbance was measured at 450 nm by using a microplate reader Bio-Rad x-Mark^TM^ spectrophotometer (Bio-Rad, Philadelphia, PA, USA) and the cell viability was calculated by comparing the viability of treated cells with that of non-treated cells, as previously described [75].

### 4.3. *Histone Deacetylase (HDAC) Activity* Assay

HDAC activity assay was performed using a colorimetric HDAC activity assay kit (Cat. K331-100; BioVision, Mountain View, CA, USA) following the manufacturer’s protocol. Briefly, nuclear extracts of HepG2 control, VPA, DOX, and the combination of VPA and DOX-treated cells were incubated with the colorimetric HDAC buffer and substrate at 37 °C for 1 h. Afterward, Lysine developer was added to stop the reaction and incubated at 37 °C for 30 min. The absorbance of HDAC was measured at the optical density (O.D) of 400 or 405 nm by using a microplate reader Bio-Rad x-Mark^TM^ spectrophotometer (Bio-Rad). The HDAC activity was normalized to the activity of control HepG2 cells and presented as a relative HDAC activity as previously described [76].

### 4.4. Hoechst Staining for Apoptotic Cell Detection

The apoptotic cell death was detected using the Hoechst 33258 (Sigma-Aldrich) nuclear staining florescence reagent [77]. The cells were grown in a 6-well plate to reach 60–70% confluence and then treated with VPA, DOX, and the VPA and DOX combination for 48 h. The cells were washed with PBS and incubated with 1 μg/mL Hoechst 33342 staining solution for 10 min. After the incubation, the cells were washed with PBS again and imaged using Nikon Eclipse TE2000-U fluorescence inverted microscopy (Nikon, Tokyo, Japan). Under the fluorescence microscope, the apoptotic cells appeared condensed and displayed fragmented nuclei.

### 4.5. Western Blot Analysis

The incubated cells were lysed using a lysis buffer (1% Triton X-100 (Sigma-Aldrich), 100 mM Tris-HCl (pH 7.5), 10% glycerol (Amresco, Solon, OH, USA), 50 mM sodium fluoride (Sigma-Aldrich), 10 mM NaCl, 1 mM phenylmethylsulfonyl fluoride (PMSF; Sigma-Aldrich), 1 mM *p*-nitrophenyl phosphate (Sigma-Aldrich), and 1 mM sodium orthovanadate (Sigma-Aldrich)) and centrifuged at 13,000 rpm at 4 °C for 15 min. The protein supernatant was quantified using the Bradford protein assay reagent (Bio-Rad), and the proteins were resolved by either 10% or 12% or 15% (for LC3B protein) sodium dodecyl sulfate polyacrylamide gel electrophoresis (SDS-PAGE). The proteins were then transferred onto nitrocellulose membranes (Bio-Rad), which were blocked with Tris-buffered 5% skimmed milk for 1 h and then incubated overnight with appropriate primary antibodies against LC3B (anti-rabbit, NB600-1384 (1:1000)) (Novus Biologicals, Littleton, CO, USA), caspase 3 (anti-rabbit, SC-7148 (1:1000)), PARP (anti-rabbit, SC-7150 (1:1000)), and actin (anti-mouse, SC-8432 (1:10,000)) (Santa Cruz Biotechnology, Dallas, TX, USA) at 4 °C. After incubation with the primary antibody, the membranes were washed three times with Tris-buffered saline supplemented with Tween 20 (TBST) at room temperature followed by a 2-h incubation with anti-mouse (SC-2005, 1:1000) or anti-rabbit (SC-2004, 1:1000) secondary antibody conjugated with horse radish peroxidase (HRP) (Santa Cruz Biotechnology). The membranes were then washed three times with TBST and the protein signals were developed using an enhanced chemiluminescence (ECL) kit (Amersham Bioscience, Piscataway NJ, USA), as described previously [78]. The intensity of proteins expression was measured and normalized by actin expression using ImageJ software (National Institute of Health, Bethesda, MA, USA).

### 4.6. Reactive Oxygen Species (ROS) Generation Analysis

Cells (1 × 10^5^ cells/well) were seeded in 12-well plates and grown overnight to confluence. The indicated cells were then pre-incubated with the ROS scavenger, NAC (1 mM). After a 1-h pre-incubation period, the cells were treated with VPA, DOX, and the VPA and DOX combination for 48 h. Intracellular ROS levels were then analyzed using the fluorescent probe H_2_DCFDA [75]. Briefly, 10 μM H_2_DCFDA was added to the cells, which were incubated for 30 min at 37 °C in the dark. The cells were then washed twice and incubated with PBS. The fluorescent images were captured using a Nikon Eclipse TE2000-U fluorescence inverted microscope (Nikon). The ROS-generating cells were counted in different fields (containing at least 40 cells per field) and calculated relative to the control group for each experimental condition.

### 4.7. AO Staining for Autophagy Detection

Cells (3 × 10^5^ cells/well) were grown overnight in six-well plates to confluence. The cells were then incubated with VPA, DOX, and the combination of VPA and DOX for 48 h with or without pre-incubation with 3-MA. After incubation, cells were treated with 5 μg/mL AO (Sigma-Aldrich, Saint Louis, MO, USA) in serum-free medium for 10 min. Then, the cells were washed twice with PBS and fluorescent images were captured by a Nikon Eclipse TE2000-U fluorescence inverted microscope (Nikon). Subsequently, AO-stained cells were counted in different fields (containing at least 40 cells per field) and presented as the percentage (%) of AO positive cells for each experimental condition [79].

### 4.8. DOX Internalization Analysis

Cells (1 × 10^5^ cells/well) were seeded in the six-well plates and grown to 60–70% confluence. For intracellular DOX measurement, the cells were incubated with VPA, DOX, and the VPA and DOX combination for 3 h. After incubation, the cells were washed with PBS to remove free and membrane-bound DOX and DOX uptake was observed in cells via fluorescent microscopy and a fluorescence microplate reader (GeminiEM, Sunnyvale, CA, USA) with excitation and emission wavelengths of approximately 470 and 570 nm, respectively. For the quantitative analysis of the internalized DOX, cells were lysed with a protein lysis buffer and fluorescence was measured using the fluorescence microplate reader (GeminiEM) with excitation and emission wavelengths of approximately 470 and 570 nm, respectively. To normalize the intracellular DOX concentrations, the DOX concentration was divided by the protein concentration, as previously described [80].

### 4.9. Determination of the Endocytosis Pathways

Cells (1 × 10^5^ cells/well) were seeded in six-well plates and grown to 60–70% confluence. To investigate the endocytosis pathway, the cells were cultured at different temperatures (37, 25 and 4 °C) in the presence of DOX (1 μM) or the VPA and DOX combination (DOX concentration, 1 μM) for 3 h. It has been established that the incubation of cells at 4 °C could block endocytosis [50]. Concurrently, cells were pre-treated for 1 h with various kinds of specific endocytosis inhibitors: CPZ (10 μM), an inhibitor of clathrin-mediated endocytosis [80]; MβCD (3 mM), an inhibitor of caveolae-mediated endocytosis [80]; and LY (20 μM), an inhibitor of macropinocytosis [71]. After incubation for 3 h with DOX or the VPA and DOX combination, the cells were washed twice with PBS and the fluorescent intensity of DOX in the cells was evaluated using the fluorescence microplate reader (GeminiEM) with excitation and emission wavelengths of approximately 470 and 570 nm, respectively. For the quantitative analysis of the internalized DOX, the cells were lysed with a protein lysis buffer and the fluorescence was measured by the fluorescence microplate reader (GeminiEM) with excitation and emission wavelengths of approximately 470 and 570 nm, respectively. To normalize the intracellular DOX concentrations, the DOX concentration was divided by the protein concentration.

### 4.10. Statistical Analysis

All experiments were conducted independently at least three times and the results were shown as the mean ± standard deviation (SD). Data were analyzed using GraphPad InStat version 3 program (Graphpad, San Diego, CA, USA). For statistical analyses, analysis of variance (ANOVA) was performed with a Bonferroni adjustment to compare the treated group with the non-treated group. A value was considered statistically significant when *p* < 0.05.

## 5. Conclusions

Overall, as an indication of the synergistic mechanism, our study demonstrated that the combination treatment of VPA and DOX was effective in the induction of cell death of HCC through the regulation of ROS and autophagy. Moreover, DOX internalization was mediated by the caveolae endocytosis pathway. Therefore, our study uncovered the potential effect of the VPA and DOX combination treatment with regard to cell death, including induction of cellular ROS generation, autophagy, and the caveolae-mediated endocytosis pathway. Our results might also indicate the potential role of the combination treatment of VPA and DOX by helping us understand their HDAC independent synergistic effect on HCC cell death through DOX internalization. These results may offer important insights into drug delivery research.

## Figures and Tables

**Table 1:** The coefficient of drug interaction (CDI) was calculated at the indicated concentration of valproic acid (VPA) and doxorubicin (DOX) by using the equation CDI = AB/(A × B). Here, AB is the ratio of the absorbance of the combination treatment group to that of the control group; A or B is the ratio of the absorbance of the single drug group to that of the control group. Hence, a CDI value <1 indicates synergism; =1 additive; or >1 antagonism. A CDI value <0.7 indicates significant synergism [44].

Order	Doxorubicin (nM)	Valproic Acid (mM)	CDI
1	10	5	0.86
2	50	5	0.84
3	100	5	0.77
4	250	5	0.16
5	500	5	0.29

## Abbreviations

VPA	valproic acid
DOX	doxorubicin
HCC	hepatocellular carcinoma
CDI	coefficient of drug interaction
PARP	poly(ADP-ribose) polymerase
ROS	reactive oxygen species
TACE	transarterial chemoembolization
HDAC	histone deacetylase
ATCC	american type culture collection
RPMI	roswell park memorial institute
DMEM	Dulbecco’s modified eagle medium
FBS	fetal bovine serum
STR	short tandem repeat
NAC	*N*-acetylcysteine
3-MA	3-methyladenine
CPZ	chlorpromazine
MβCD	methyl-β-cyclodextrin
LY	LY294002
CD	cyclodextrin
AO	acridine orange
H_2_DCFDA	2′,7′-dichlorodihydrofluorescein diacetate
PBS	phosphate-buffered saline
PMSF	phenylmethylsulfonyl fluoride
SDS-PAGE	sodium dodecyl sulfate polyacrylamide gel electrophoresis
TBST	tris-buffered saline tween 20
HRP	horse radish peroxidase
ECL	enhanced chemiluminescence
SD	standard deviation
ANOVA	analysis of variance
HDEI	histone deacetylase inhibitor
ATC	anaplastic thyroid carcinoma
GSH	glutathione
